# Enhanced Cysteinyl-Leukotriene Type 1 Receptor Expression in T Cells from House Dust Mite-Allergic Individuals following Stimulation with Der p

**DOI:** 10.1155/2015/384780

**Published:** 2015-03-31

**Authors:** Maryse Thivierge, Sylvie Turcotte, Marek Rola-Pleszczynski, Jana Stankova

**Affiliations:** Division of Immunology and Allergy, Department of Pediatrics, Faculty of Medicine and Health Sciences, Université de Sherbrooke, Sherbrooke, QC, Canada J1H 5N4

## Abstract

In order to determine the potential for allergen to modulate T cell expression of the CysLT_1_ receptor and responsiveness to leukotrienes, peripheral blood mononuclear cells from house dust mite-allergic or nonallergic individuals were incubated with* D. pteronyssinus *allergen (Der p). Baseline CysLT_1_ expression was similar in both groups of donors, but Der p significantly enhanced CysLT_1_ expression in CD4^+^ and CD8^+^ T cells of only allergic individuals and induced enhanced responsiveness of CD4^+^ T cells to LTD_4_ in terms of calcium mobilisation. This effect was prevented by the CysLT_1_ antagonist MK571. Der p also induced IL-4 and IL-10 production, and neutralizing antibody to IL-4 prevented both the enhanced CysLT_1_ expression and the enhanced responsiveness of T cells to LTD_4_ induced by Der p. In allergic individuals, Der p also induced T cell proliferation and a Th2-biased phenotype. Our data suggest that, in allergen-sensitized individuals, exposure to allergen can enhance T cell expression of CysLT_1_ receptors through a mechanism involving IL-4 production. This, in turn, would induce CD4^+^ T cell responsiveness to cysteinyl-leukotrienes and Th2 cell activation.

## 1. Introduction

Cysteinyl-leukotrienes (cysLTs), leukotriene C_4_, LTD_4_, and LTE_4_, are lipid mediators of inflammation well known to be involved in the pathogenesis of asthma and the resulting pulmonary inflammation [1]. They are mainly produced by eosinophils, mast cells, basophils, monocytes, macrophages, and dendritic cells (DC) from arachidonic acid through the 5-lipoxygenase pathway [2]. CysLTs act on at least three G-protein-coupled receptors designated CysLT_1_, CysLT_2_, and GPR99 [3–6]. CysLT_1_, recognized as the high-affinity receptor for LTD_4_, is expressed mainly in peripheral blood leukocytes, including eosinophils, monocytes, neutrophils, basophils, DC, B lymphocytes, and T cells, as well as in mast cells, interstitial lung macrophages, and bronchial smooth muscle cells [7–13]. CysLT_2_ binds LTC_4_ and LTD_4_ with similar affinity and is widely expressed in many tissues, including heart, adrenal, lung, spleen, endothelium, and peripheral blood leukocytes, and less strongly in the brain [5]. In contrast, the more recently characterized GPR99 is mainly activated by LTE_4_ and is widely expressed in nonhematopoietic tissues [6]. The expression of CysLT_1_ and CysLT_2_ receptors can be modulated in the presence of cytokines such as IL-4, IL-5, IL-13, IFN-*γ*, or TGF-*β*, at both the mRNA and protein levels in different cell populations [12, 14–17]. CysLTs have been found to promote cytokine and chemokine expression in various cellular models and IgE production by human B cells [15, 18–21].

House dust mites (HDM) are a major source of allergens that contribute to the rising incidence of allergic asthma [22]. Asthma and allergic rhinitis are inflammatory diseases characterized by the influx of multiple cell types to affected tissue sites and the increased expression of both CysLT_1_ and CysLT_2_ as compared with circulating cells or those present in noninflamed tissue [7, 23]. These diseases are associated with a complex cytokine milieu described mainly as Th2 in nature (IL-4, IL-13, and IL-9), but including Th1 cytokines (IFN-*γ*) as well. Exposure to mite allergens induces cysLT production from a number of constitutive and inflammatory cells in asthmatic airways, including epithelial cells, mast cells, and eosinophils [24]. In atopic patients, DC pulsed with HDM allergens produced a significant increase in cysLT production and showed a Th2-favoring phenotype with a Th2-skewed cytokine production from autologous CD4^+^ T cells [25]. In mice, HDM allergen was shown to elicit both cysLT generation and CysLT_1_ receptor-mediated priming of dendritic cell function in an autocrine fashion to promote allergic inflammation [26]. Different studies have shown that DC from allergic patients exposed to the allergen* Dermatophagoides pteronyssinus* (Der p) were able to promote Th2 responses through an overproduction of IL-10 and to favor an increase in IL-4 production by autologous T cells [27, 28]. The proteolytic activity of Der p has been shown to bias human T cells towards a helper type 2 cytokine profile by inducing them to produce more IL-4 and less IFN-*γ* [29]. Such findings suggest that exposure to an allergen induces T helper cell polarization to Th2 in the sensitized host.

Whereas resting T lymphocytes were found to display low cell surface expression of CysLT_1_ and CysLT_2_, T cell activation through the T cell receptor (TcR) was shown to induce an important rise in the percentages of CysLT_1_ and CysLT_2_ positive cells [30]. In addition, CysLT_1_ upregulation after TcR activation of mouse T cells was associated with enhanced LTD_4_-elicited calcium flux and migration toward LTD_4_ [13].

In the present study, we investigated the effects of Der p on CysLT receptor expression on human T cell populations. In particular, we incubated PBMC from HDM-allergic or HDM-nonallergic individuals with Der p allergen and analyzed the expression and functional activity of CysLT_1_ and CysLT_2_ on T cell subsets. We hypothesized that Der p could differentially modulate these receptors according to the allergic status of the donors.

## 2. Materials and Methods

### 2.1. Reagents

The HDM allergen* Dermatophagoides pteronyssinus*, Der p, was obtained from Omega Laboratories (Pointe-Claire, QC, Canada). Human recombinant IL-4 and IL-10 were purchased from Peprotech (Rocky Hill, NJ, USA). LTD_4_ and rabbit polyclonal anti-human CysLT_1_ and CysLT_2_ Ab were obtained from Cayman Chemical (Ann Arbor, MI). The CysLT_1_ antagonist montelukast was a generous gift from Merck-Frosst (Point-Claire, Québec, Canada) whereas MK-571 was obtained from Biomol Research Laboratories (Plymouth Meeting, PA). Rabbit IgG isotype control was purchased from Southern Biotechnology Associates (Birmingham, AL); mouse IgG isotype control (G155-178), mouse anti-CD4 PE-CY5, mouse anti-CD8 PE, and anti-CD3 APC were purchased from BD Pharmingen (Mississauga, Ontario, Canada). Rabbit polyclonal anti-human IL-4 and IL-10 Ab were purchased from R&D Cedarlane.

### 2.2. Cell Culture

Venous blood was collected from donors sensitive or not sensitive to the HDM allergen Der p by history and skin tests or specific serum IgE. All donors were recruited following consent to a protocol (98-20-R16) approved by the Université de Sherbrooke Ethics Review Board. Peripheral blood mononuclear cells (PBMC) were isolated through Ficoll-Hypaque density centrifugation. PBMC were used for some experiments or depleted of their monocyte population by adherence. CD4^+^ and CD8^+^ T cells were purified from whole blood lymphocytes by depletion of contaminating cells using the “human CD4^+^ T or CD8^+^ T cell enrichment kit, negative selection” (Stemcell Technologies, Vancouver, BC, Canada) following the manufacturer's instructions. Purity was greater than 98%. The cells were washed and resuspended in complete RPMI 1640 medium supplemented with 5% FBS and maintained at 37°C in 5% CO_2_. Cells were stimulated with the cytokines IL-4 (40 ng/mL) or IL-10 (20 ng/mL) for 48 h before RNA was harvested or 72 h for studies involving protein expression or calcium flux assay. In some experiments, PBMC were stimulated with vehicle control (glycerin) or Der p (200 AU/mL) for 48 to 72 h before supernatant collection and CD4^+^ and CD8^+^ T cell subpopulations analysis for their CysLT1R expression or responsiveness. In selected experiments, total lymphocytes were stimulated with IL-4 and IL-10 or incubated on anti-CD3-precoated petri dishes.

### 2.3. Flow-Cytometric Measurement of Expression of CysLT Receptors

Following treatment, PBMC were washed, resuspended with PBS, fixed with 2% paraformaldehyde for 15 min at room temperature, and permeabilized with 0.1% saponin for an additional 20 min at room temperature. After blocking with human IgG (20 *μ*g/1 × 10^6^ cells) for 15 min at room temperature, cells were resuspended with PBS-2% FBS and labeled for 30 min with rabbit polyclonal anti-human CysLT_1_ or CysLT_2_ Abs or with rabbit IgG isotype control Ab. Cells were then washed with PBS and incubated for 30 min with FITC-conjugated goat anti-rabbit IgG. Cells were also labeled with mouse anti-human CD4 PE-CY5, anti-CD8 PE, and anti-CD3APC (BD Pharmingen). Finally, cells were acquired and analyzed on a FACSCalibur flow cytometer using the CellQuest Pro software.

### 2.4. Assessment of T Cell Proliferation

PBMC were washed twice with PBS, incubated in protein-free PBS, and labeled for 5 min at room temperature with 5 *μ*M of CSFE (5-carboxy fluorescein diacetate succinimidyl ester) (Molecular Probes, Eugene, OR). Cells were washed three times with PBS containing 5% FBS, resuspended in RPMI 5% FBS, and incubated with Der p (200 AU/mL) or its vehicle (glycerin) for up to 8 days. At different periods of time, cells were collected and labelled with anti-CD4 PE-Cy5, anti-CD8 PerCP-Cy5, and anti-CD3 APC (BD Pharmingen). Fractions of stained cells were acquired and analyzed on a FACSCalibur flow cytometer to calculate the percentage of CD4^+^ T cells undergoing division. A CFSE profile was generated using FlowJo v7/8 (Tree Star Inc., Ashland, OR, USA).

### 2.5. Th1 and Th2 Marker Analysis

PBMC were incubated in RPMI 5% FBS with Der p (200 AU/mL) or its vehicle (glycerin) for up to 11 days. On day 6, one volume of fresh medium supplemented with the stimuli was added to the cells. On day 11, cells were harvested and incubated for 30 minutes at 4°C with fluorochrome-conjugated monoclonal antibodies: CD184 PE (CXCR4 Ab), CD183 Alexa 488 (CXCR3 Ab), and CD294 Alexa 647 (CRTH2 Ab) (BD Pharmingen, San Diego, CA, USA) antibodies known to recognize Th cell surface markers associated with Th0, Th1, and Th2, respectively [31, 32]. Labeled cells were resuspended in PBS and fluorescence was analyzed on a FACSCalibur cytometer equipped with CellQuest Pro software.

### 2.6. RNA Isolation and Real-Time Quantitative PCR

Cellular RNA was obtained using Trizol reagent (Invitrogen, Burlington, ON, Canada) according to the manufacturer's instructions. After total RNA purification with RNeasy kit (Qiagen, Mississauga, ON, Canada), 1.0 *μ*g of RNA was converted to cDNA with oligo-dT (Fermentas, Burlington, ON, Canada) and reverse transcriptase (M-MLV; Promega, Madison, WI, USA) in a volume of 20 *μ*L. CysLT_1_, CysLT_2_, and GAPDH expression was measured using real-time quantitative PCR performed on a Rotor-Gene 3000 (Corbett Research, Kirkland, QC, Canada). The following oligonucleotide primer sets were obtained from Integrated DNA Technologies (Coralville, IA, USA): human CysLT_1_: forward, 5′-CCTCAGCACCTATGCTTTGT-3′ and reverse, 5′-ATTGTCTTGTGGGGGCTCAA-3′ (amplifying a 249-bp fragment); human CysLT_2_: forward, 5′-AGACTGCATAAAGCTTTGGTTATC-3′ and reverse, 5′-ATACTCTTGTTTCCTTTCTCAACC-3′ (amplifying a 196-bp fragment); and human GAPDH: forward, 5-GATGACATCAAGAAGGTGGTGAA-3 and reverse, 5-GTCTTACTCCTTGGAGGCCATGT-3 (amplifying a 246-bp fragment).

Each sample for real-time PCR consisted of 1 *μ*L cDNA, 2.5 mM MgCl_2_, 100 *μ*M dNTP, 1 *μ*M of primers, 2.5 *μ*L of 10x PCR buffer, 0.5 unit of Taq polymerase (New England Biolabs, Pickering, Ontario, Canada), and 0.8 *μ*L of SYBR Green (Molecular Probe, Eugene, OR; 1/1000 stock dilution) in a reaction volume of 25 *μ*L. The cycling program consisted of an initial denaturation at 95°C for 5 min and 40 cycles of amplification conditions as follows: 95°C (30 sec), 60°C (30 sec), and 72°C (30 sec), with the fluorescence read at the end of each cycle. Comparison of the expression of each gene between its control and stimulated states was determined with the delta-delta (ΔΔ)Ct, according to the following formula: ΔΔCt = [(Ct G.O.I.Ctl − Ct HK.G.Ctl) − (Ct G.O.I.STIM. − Ct HK.G.STIM.)]. Results were then transformed into fold variation measurements: fold increase = 2^ΔΔCt^.

### 2.7. Measurement of Intracellular Calcium

Intracellular free calcium was measured on a FACSCalibur (Becton-Dickinson). T cells at 4 × 10^6^/mL were incubated for 30 min at room temperature in 1 mL HBSS containing 0.35 g/L NaHCO_3_ and 10 mM HEPES pH 7.0, with 3 *μ*M fluo-4AM (Molecular Probes). The dye-loaded cells were then washed and resuspended in HBSS containing 2 mM CaCl_2_ and baseline fluorescence was measured at 0 s. Fluorescence released after LTD_4_ stimulation (100 nM) was recorded every 15 s for 2 min. Intracellular free calcium was measured on a FACSCalibur flow cytometer (Becton-Dickinson) using the CellQuestPro software (BD Bioscience) and analyzed with FlowJo v7/8 (Tree Star Inc., Ashland, OR, USA).

### 2.8. Cytokine Detection

Cytokines were measured with commercial ELISA (BD Biosciences, San Diego, CA, USA) according to the manufacturer's instructions. Lower limits of detection for these ELISA were 2 pg/mL for both IL-4 and IL-10.

### 2.9. Statistical Analysis

Statistical significance was calculated using Prism 5 software (GraphPad Software, San Diego, CA). For analysis of differences between experimental groups, Student's *t*-test and one-way or two-way ANOVA with Bonferroni posttest were used, as appropriate. Values of *P* ≤ 0.05 were considered statistically significant.

## 3. Results

### 3.1. CysLT_1_ Expression on T Cells Is Enhanced by Der p

**Figure 1:**
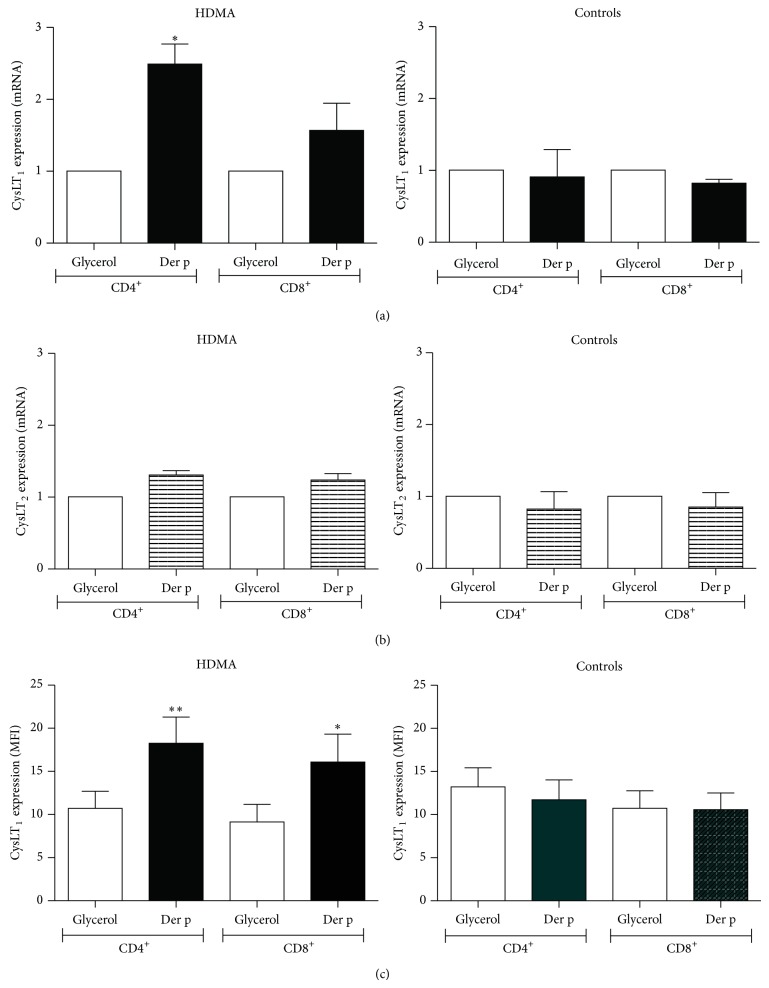
Der p effect on T cell CysLT_1_ and CysLT_2_ mRNA and protein expression. Comparison of CysLT_1_ and CysLT_2_ mRNA expression in CD4^+^ and CD8^+^ T cells from healthy controls and HDM-allergic (HDMA) patients following stimulation with the Der p allergen (200 AU/mL). PBMC from healthy control and HDMA subjects were cultured for 48 h (qPCR) or 72 h (FACS) in the presence of glycerol vehicle or Der p before CD4^+^ and CD8^+^ T cells were purified and collected for analysis. CysLT_1_ (a) and CysLT_2_ (b) mRNA expression was measured by real-time quantitative PCR analysis. Data are presented as fold (ΔΔCt) increases over GAPDH mRNA (±SEM). ^∗^ *P* < 0.05 and ^∗∗^ *P* < 0.01, relative to vehicle glycerol; *n* = 6 for controls; *n* = 10 for HDMA. Cell surface expression of CysLT_1_ (c) receptor was evaluated using rabbit polyclonal anti-CysLT_1_ receptor Ab, followed by labeling with FITC-conjugated goat anti-rabbit IgG. Cells were further incubated with anti-CD4 PE-Cy5 and anti-CD8 PE Ab before analysis on a FACSCalibur flow cytometer. Data are expressed as geometric mean (±SEM) fluorescence intensity (MFI). ^∗^ *P* < 0.05; *n* = 6 for controls; *n* = 10 for HDMA.

In a first series of experiments, we compared the effect of Der p on CysLT_1_ and CysLT_2_ expression in T cells from HDM-allergic and HDM-nonallergic individuals. PBMC from individuals of either group were exposed to either glycerin vehicle or Der p (200 AU/mL) for 48 h before purification of CD4^+^ and CD8^+^ T cells. RNA from these purified cells was then isolated and CysLT_1_ and CysLT_2_ mRNA expression was analyzed by real-time PCR. As illustrated in Figures 1(a) and 1(b), CD4^+^ and CD8^+^ T cells from HDM-allergic subjects showed a significantly increased CysLT_1_, but not CysLT_2_, mRNA expression upon stimulation with Der p. In contrast, Der p failed to modulate either CysLT_1_ or CysLT_2_ mRNA expression in cells from nonallergic individuals.

In additional experiments, PBMC were also exposed for 72 h to either Der p or glycerin vehicle before cytometry analysis of CysLT_1_ or CysLT_2_ protein expression on CD4^+^ and CD8^+^ T cell subpopulations. Whereas both receptors are widely expressed on peripheral blood leukocytes, they are not highly expressed on circulating T cells, with less than 10% of cells expressing CysLT_1_ or CysLT_2_ [5, 7, 23]. Cell surface CysLT_1_ and CysLT_2_ expression was constitutively present on both subpopulations of T cells with basal levels of CysLT_1_ and CysLT_2_ expression ranging from 2.5% to 10% of cells and not significantly different between healthy donors and HDM-sensitive subjects (data not illustrated). However, stimulation with Der p significantly increased CysLT_1_ expression (Figure 1(c)), without affecting CysLT_2_ expression (not shown), in both T cell subpopulations from HDM-allergic donors. In contrast, as observed at the mRNA level, CysLT_1_ expression on T cells from nonallergic donors was not modulated by Der p exposure.

### 3.2. Proliferation and Polarization of T Cells

**Figure 2:**
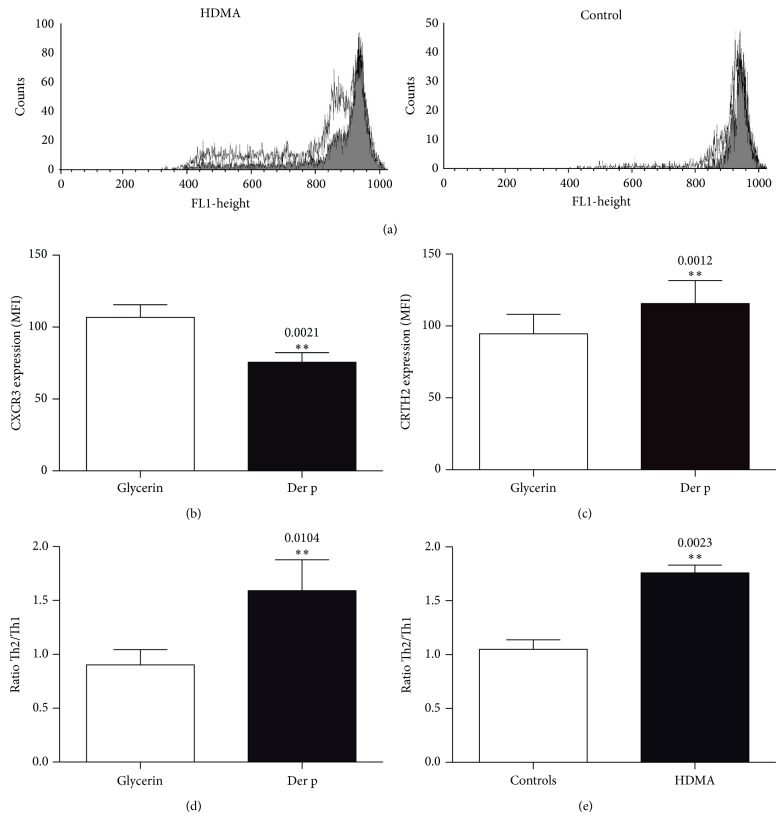
Flow-cytometric analysis of T cell proliferation and Th cell polarization. CFSE-labeled CD4^+^ T cells from HDM-allergic or HDM-nonallergic individuals were cultured with Der p or glycerin as described in Section 2. T cell division was analyzed by flow cytometry and illustrated as a CFSE division profile (a). Dead cells were excluded based on their light scattering properties. Nondividing CD4^+^ T cells in the absence of allergen are shown as dark grey histograms. CD4^+^ T cells that have divided in response to Der p are shown in light grey histograms, based on CFSE dilution peaks. One representative experiment of three is illustrated.* In vitro* polarization of human Th1 and Th2 precursors following 11 days of culture with glycerin or Der p was determined by flow cytometry using Alexa-conjugated Abs for human CXCR3 (b) or CRTH2 (c), respectively. Data are expressed as geometric mean (±SEM) fluorescence intensity (MFI). *P* values are indicated. HDMA = house dust mite-allergic donors. Th2/Th1 ratio of CD4^+^ T cells from HDM-allergic donors following 11 days of culture with glycerin or Der p (d), *n* = 6; Th2/Th1 ratio of Der p-stimulated CD4^+^ T cells from nonallergic and HDM-allergic individuals (e), *n* = 6 for controls; *n* = 10 for HDMA.

T cell polarization toward a Th1 or a Th2 profile is dependent on the cytokines present when the interaction of APC with T cells occurs. Allergic diseases are characterized by a predominant Th2 profile. We thus examined whether Der p could induce T cells from HDM-allergic individuals to proliferate and to develop a Th2 phenotype. T cell proliferation was measured by CFSE dye dilution. As depicted in Figure 2(a), the proliferative response of CFSE-labeled CD4^+^ T cells from HDM-allergic patients was enhanced following Der p stimulation of PBMC. In contrast, we observed no proliferation of CD4^+^ T cells from nonallergic donors.

We next examined the expression of markers that have been associated with either Th1 or Th2 cell types on CD4^+^ and CD8^+^ cells from allergic patients and healthy individuals. CXCR3 (CD183) has been proposed as a marker associated with Th1 responses [31] and the PGD_2_ receptor CD294, also referred to as chemoattractant receptor expressed on Th2 cells (CRTH2), has recently emerged as a marker of Th2-cell functions [32]. In the present study we investigated the effect of Der p on the expression of these two markers on CD4^+^ T cells from HDM-sensitive and HDM-nonallergic donors. Using flow cytometry analysis, the expressions of CD183 (Th1 marker) and CD294 (Th2 marker) were assessed in CD4^+^ T cells after incubation of PBMC in the presence of Der p. As illustrated in Figures 2(b)–2(e), Der p treatment significantly reduced the proportion of CD183 positive cells and increased the proportion of CD294 positive cells in HDM-allergic donors, with a ratio of Th2/Th1 cell markers greater than 1.5. Nonallergic donors showed an unchanged ratio of approximately 1.0.

### 3.3. Role of IL-4 in Der p-Enhanced CysLT_1_ Expression on T Cells

**Figure 3:**
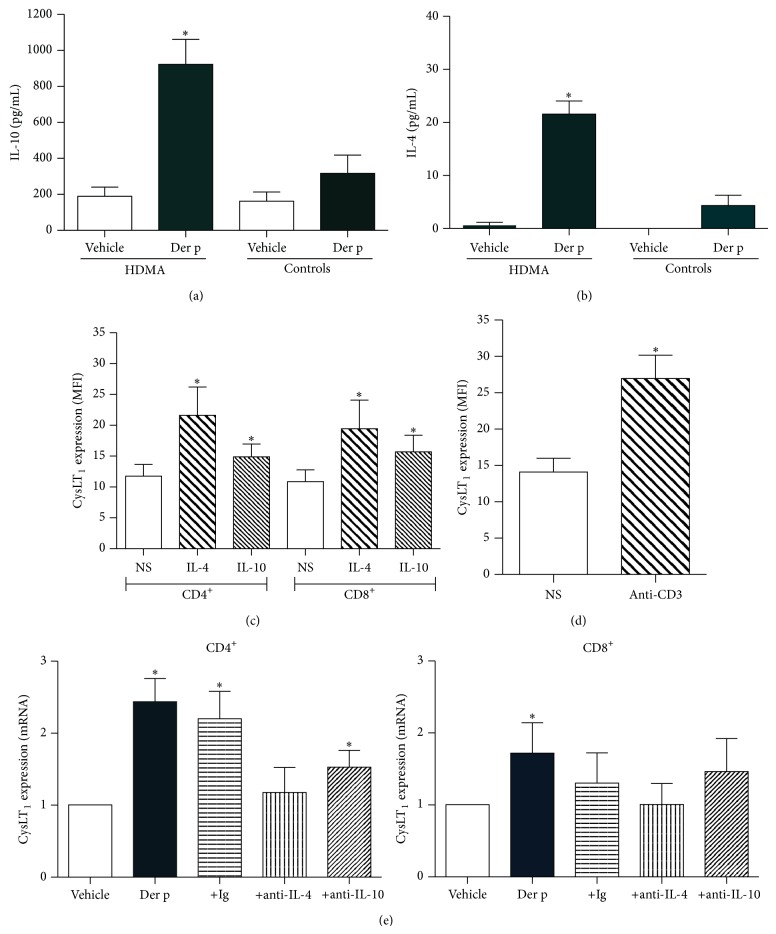
Cytokine production and effects in HDM-allergic and HDM-nonallergic individuals. PBMC from healthy controls and HDMA subjects were cultured for 72 h in the presence of glycerol vehicle or Der p before supernatants were collected for analysis. IL-4 (a) and IL-10 (b) production in cell-free culture supernatants were measured by ELISA. Results are expressed as means ± SEM. *n* = 5. CysLT_1_ expression by T cells following stimulation with IL-4 (40 ng/mL) or IL-10 (20 ng/mL) (c) for 48 h or following TcR/CD3 stimulation (d) for 72 h was measured by flow cytometry. Data are expressed as geometric mean (±SEM) fluorescence intensity (MFI). (e) PBMC from HDM-allergic (HDMA) individuals were cultured for 48 h or 72 h with Der p in the presence of neutralizing anti-IL-4 or anti-IL-10 Abs or control IgG. CysLT_1_ mRNA expression by CD4^+^ T cells and CD8^+^ T cells purified from PBMC at 48 h after stimulation was measured by real-time quantitative PCR analysis. Data are presented as fold (ΔΔCt) increases over GAPDH mRNA ± SEM for *n* = 5 experiments; ^∗^ *P* < 0.05 with respect to the vehicle.

We evaluated the effect of Der p on the production of IL-4 and IL-10 by PBMC from allergic patients and healthy donors. Cell-free supernatants were collected from PBMC incubated for 48 h with Der p and analyzed for cytokine production by ELISA. PBMC from HDM-sensitive patients produced significantly higher amounts of the cytokines IL-10 and IL-4 (Figures 3(a) and 3(b)). In contrast, when PBMC from HDM-nonallergic donors were incubated with Der p, the induction of IL-4 and IL-10 production was only modest and statistically nonsignificant. Basal IL-4 and IL-10 production by unstimulated cells was similar in PBMC from allergic and healthy subjects.

We next tested the effect of IL-4 and IL-10 on CysLT_1_ expression in T cells. CD4^+^ T cells from normal donors were stimulated with anti-CD3 Ab or with the cytokines IL-4 and IL-10 for 72 h. Stimulation of both CD4^+^ and CD8^+^ cells with either IL-4 or IL-10 resulted in enhanced CysLT_1_ expression (Figure 3(c)). Similarly, T cell activation through the T cell receptor/CD3 complex was found to enhance CysLT_1_ expression in T cells (Figure 3(d)).

We next investigated whether the Der p-induced effect on CysLT_1_ expression was dependent on IL-4 and/or IL-10. To this aim, we added anti-IL-4 or anti-IL-10 neutralizing Ab to the PBMC from allergic donors before stimulation with Der p and measured CysLT_1_ expression in CD4^+^ and CD8^+^ T cells after 48 h. As shown in Figure 3(e), addition of anti-IL-4 Ab significantly abrogated the effect of Der p on CysLT_1_ mRNA expression of both CD4^+^ and CD8^+^ cells, whereas the use of anti-IL-10 only partially reversed this effect. A similar effect of these anti-cytokine Abs was also observed at the CysLT_1_ protein level (data not illustrated).

### 3.4. Functional Analysis of Increased Leukotriene Receptor Expression

**Figure 4:**
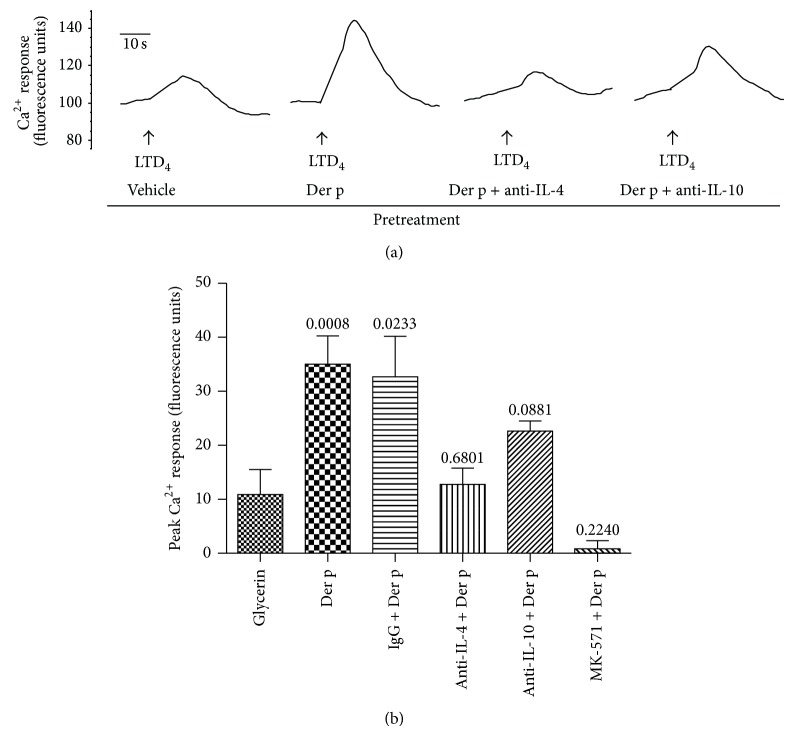
Functional analysis of increased CysLT_1_ expression on T cells. The biological significance of the enhanced CysLT_1_ receptor expression on T cells was assessed by measuring intracellular calcium flux in response to LTD_4_. PBMC from HDM-allergic (HDMA) individuals were exposed for 72 h to Der p in the presence or absence of neutralizing Abs to IL-4 or IL-10 or of the CysLT_1_ receptor antagonist MK571. Cells were then labeled with anti-CD3 and anti-CD4 Ab and loaded with Fluo4-AM for the calcium mobilization assay on a FACSCalibur flow cytometer. (a) Raw data representative of 4 separate experiments. (b) Compiled data of peak calcium responses. *n* = 3; *P* values indicated above histograms, with respect to the vehicle.

Having demonstrated that Der p and IL-4 can increase CysLT_1_ expression on T cells, we investigated whether the increased levels of expression led to enhanced LTD_4_ signaling in T cells. The biological activity of the receptors on T cells was assessed by measuring intracellular calcium flux in response to LTD_4_, the physiological ligand of CysLT_1_. PBMC from allergic and nonallergic subjects were exposed for 72 h to Der p, after which cells were analyzed in a calcium mobilization assay. As shown in Figures 4(a) and 4(b), stimulation of CD4^+^ T cells from HDM-allergic donors with LTD_4_ induced a discrete response in calcium mobilization, and pretreatment with Der p resulted in an enhanced response to the ligand. The Ca^2+^ mobilization observed in CD4^+^ T cells was prevented by MK571, a specific CysLT_1_ antagonist. Furthermore, the addition of neutralizing Abs to IL-4 abrogated the enhanced calcium response to LTD_4_ of Der p-treated CD4^+^ T cells from HDM-allergic individuals. In contrast, neutralizing Abs to IL-10 failed to affect the response. Whereas CD4^+^ T cells from nonallergic donors responded to LTD_4_ stimulation similarly to HDM-allergic donors in terms of calcium mobilization, they maintained the same basal responsiveness whether or not they had been preincubated with Der p (data not illustrated).

## 4. Discussion

The present study demonstrated that the Der p allergen induced an increased expression of CysLT_1_ receptor in CD4^+^ and CD8^+^ T cells from HDM-allergic subjects, whereas Der p had no significant effects on T cells from non-HDM-allergic individuals. This upregulation of CysLT_1_ expression by Der p induced an enhanced responsiveness of T cells to LTD_4_. Previous studies reported that HDM allergens could modulate cysLT production or CysLT receptor expression in mouse and human dendritic cells (DC). Hence, in murine bone marrow-derived DC, Machida et al. [26] reported that* D. farinae* allergen significantly increased CysLT_1_ receptor, 5-LO, FLAP, and LTC_4_S mRNA and cysLT production. Saeki et al. [25] reported that human monocyte-derived DC from* D. farinae* allergen-sensitized subjects expressed CysLT_1_ receptor, 5-LO, FLAP, and LTC_4_S mRNA and* D. farinae* pulsing significantly enhanced their cysLT production. A link between HDM allergens and cysLT production also has been reported in other cell populations of asthmatic airways, including epithelial cells, mast cells, and eosinophils [24, 33].

In the present study, the effect of Der p on T cell expression of CysLT_1_ was associated with an enhanced production of IL-4 and IL-10 in PBMC from allergic subjects, and both the enhanced CysLT_1_ expression and the enhanced responsiveness of T cells to LTD_4_ could be prevented when endogenous IL-4, but not IL-10, was neutralized. IL-4 and IL-10 were also able to significantly enhance mRNA and protein levels of CysLT_1_ in purified T cells. In contrast, T cells from non-HDM-allergic individuals appeared to be unable to respond to Der p suggesting that this effect would be antigen specific and dependent on prior sensitization. However, augmentation in CysLT_1_ expression could be induced in T cells from nonallergic donors through activation of the T cell receptor/CD3 complex or with the cytokines IL-4 and IL-10.

Previous studies have indicated that CysLT receptor expression could be altered by various stimuli in different cell types. Others and we have previously reported the expression of CysLT_1_ receptors on leukocytes, including alveolar and monocyte-derived macrophages, as well as DC and B lymphocytes [3, 7, 8, 10, 17]. In particular, CysLT_1_ expression levels were upregulated by IL-13 and IL-4 in monocytes and macrophages and resulted in enhanced Ca^2+^ transients and chemotactic responses to LTD_4_ [17]. Whereas T lymphocytes were previously shown to display low surface expression of CysLT_1_ and CysLT_2_, T cells activation through the T cell receptor (TcR) was shown to enhance the percentages of CysLT_1_ and CysLT_2_ positive cells [30]. In a murine model, Prinz et al. [13] reported CysLT_1_ upregulation after TcR activation of mouse T cells which was associated with enhanced LTD_4_-elicited calcium flux and migration toward LTD_4_. More recently, IL-4 was also shown to upregulate CysLT_1_ and CysLT_2_ expression on T and B cells, whereas IFN-*γ* was shown to induce CysLT_2_ expression on monocytes and T and B lymphocytes [12]. IL-4 is a known activator of STAT6 [34]. The identification of a STAT6 response element in the CysLT_1_ receptor promoter was proposed to be one of the mechanisms that mediate enhanced CysLT_1_ expression following IL-4 stimulation [35]. Our observation that Der p induced CysLT_1_ expression in T cells could be partially dependent on this mechanism of transactivation of the CysLT_1_ promoter by STAT6.

The polarization of the immune response toward a Th2 or a Th1 profile can be mediated by APC following antigen presentation and interaction with T cells. In sensitized individuals, recruitment of Th2 cells and subsequent production of Th2-type cytokines like IL-4, IL-5, and IL-13 orchestrate the inflammatory response to inhaled aeroallergens. Our results showed that Der p induced CD4^+^ T cells to proliferate and favored T lymphocyte differentiation towards a Th2 phenotype in HDM-allergic donors. Hence, we observed that Der p treatment induced IL-4 production and increased the expression of CRTH2 (CD294), a marker for Th2 cells [32], while it reduced the expression of CXCR3 (CD183), a marker for Th1 cells [31]. In counterpart, T cells from healthy control donors did not proliferate in the presence of Der p and their Th1 and Th2 markers were not affected. These observations are in concordance with several studies demonstrating the promotion of Th2 responses by HDM allergens. Mite allergen-specific T cell clones from atopic donors exhibit a Th2 cytokine profile [36] and require IL-4 for their optimal growth [37]. Different studies have shown that DC from allergic patients exposed to the allergen Der p 1 could promote Th2 responses. In atopic patients, DC pulsed with HDM allergens produced a significant increase in cysLT production and showed a Th2-favoring phenotype with a Th2-skewed cytokine production from autologous CD4^+^ T cells [25]. Also Der p 1 induced a rapid and higher production of proinflammatory cytokines TNF-*α*, IL-1*β* and the type 2 cytokine IL-10 by DC from HDM-sensitive patients and that their purified T cells stimulated by autologous Der p 1-pulsed DC preferentially produced IL-4 rather than IFN-*γ* [27, 28]. In another study, Der p 1 was shown to bias human T cells towards a type 2 cytokine profile by inducing them to produce more IL-4 and less IFN-*γ* [29]. DC from allergic patients exposed* in vitro* to Der p 1 were shown to rapidly increase their TARC (CCL17) and MDC (CCL22) production, two type-2 attracting chemokines known to be involved in the polarisation of the immune response [38]. DC from patients sensitive to* Dermatophagoides* react to Der p 1 (IL-6 and IL-10 production) differently from DC from healthy donors (IL-12 production), not only* in vitro* [27, 28], but also* in vivo* [39]. It has been reported that HDM extracts could also stimulate the production of IL-4 and IL-13 in mite-sensitive asthmatic basophils and activate IL-8 release in epithelial cells [40, 41]. IL-10 and IL-4 production was shown to be induced by peripheral blood leukocytes from patients with asthma after exposure to the mite allergen* D. farinae* [42].

Our findings are also in concordance with recent work of Parmentier et al. which showed that human Th2 cells preferentially express* CYSLTR1* mRNA after* in vitro* differentiation from naive precursors and selectively respond to cysLTs with calcium flux and chemotaxis [11].

Proteases, including serine and cysteine protease, have been considered critical factors in the cytokine expression modulated by mite allergens [43, 44]. Der p 1 protein, the major allergen from the mite* Dermatophagoides pteronyssinus*, is characterized by its cysteine protease activity. However, studies have demonstrated that cytokine production induced by mite extracts is associated with both protease-independent and protease-mediated mechanisms. Der p 1 has been shown to induce the release of GM-CSF, IL-6, and IL-8 because of its proteolytic activity in bronchial epithelial cells [45, 46]. The proteolytic activity of Der p 1 has also been shown to favor human T cell production of more IL-4 and less IFN-*γ* [29]. In THP-1 cells,* Dermatophagoides pteronyssinus* strongly increased the release of MCP-1, IL-6, and IL-8 and these responses were not associated with serine and cysteine proteases [47]. In our study, we used the cysteine protease-specific inhibitor E-64 alone and together with the serine protease inhibitor aprotinin during the incubation of PBMC with Der p 1. We observed only partial diminutions in Der p-induced CysLT_1_ mRNA expression and calcium mobilization in CD4^+^ T cells with the combination of the two inhibitors (data not illustrated).

In conclusion, whereas CysLT receptor expression has been generally reported to be low in T cells, allergen-induced inflammation could activate T cells and enhance their expression of the receptors, thus making them more responsive to cysLTs present in the tissues. More precisely, our data suggest that, in allergen-sensitized individuals, exposure to allergen can enhance T cell expression of CysLT_1_. This, in turn, would induce enhanced CD4^+^ T cell responsiveness to cysLTs, T cell activation, and Th2 polarization. These findings identify a novel mechanism by which potent indoor allergens may activate immune cells to promote allergic inflammation.

## Abbreviations

HDM: House dust mite
LT: Leukotriene
cysLT: Cysteinyl-leukotriene
CysLT: cysLT receptor
PBMC: Peripheral blood mononuclear cells
Th2: T helper 2
Der p: * Dermatophagoides pteronyssinus*.
